# Epstein–Barr Virus in Gastro-Esophageal Adenocarcinomas – Single Center Experiences in the Context of Current Literature

**DOI:** 10.3389/fonc.2015.00073

**Published:** 2015-03-26

**Authors:** Vera Genitsch, Alexander Novotny, Christian A. Seiler, Dino Kröll, Axel Walch, Rupert Langer

**Affiliations:** ^1^Institute of Pathology, University of Bern, Bern, Switzerland; ^2^Department of Surgery, Klinikum Rechts der Isar, Technische Universität München, München, Germany; ^3^Department of Visceral Surgery and Medicine, Inselspital University Hospital Bern, University of Bern, Bern, Switzerland; ^4^German Research Center for Environmental Health, Institute of Pathology, Helmholtz Zentrum München, Neuherberg, Germany

**Keywords:** EBV, esophageal adenocarcinoma, gastric cancer, adenocarcinoma of the gastro-esophageal junction, EBER

## Abstract

Epstein–Barr virus (EBV)-associated gastric carcinomas (GC) represent a distinct and well-recognized subtype of gastric cancer with a prevalence of around 10% of all GC. In contrast, EBV has not been reported to play a major role in esophageal adenocarcinomas (EAC) and adenocarcinomas of the gastro-esophageal junction (GEJ). We report our experiences on EBV in collections of gastro-esophageal adenocarcinomas from two surgical centers and discuss the current state of research in this field. Tumor samples from 465 primary resected gastro-esophageal adenocarcinomas (118 EAC, 73 GEJ, and 274 GC) were investigated. Presence of EBV was determined by EBV-encoded small RNAs (EBER) *in situ* hybridization. Results were correlated with pathologic parameters (UICC pTNM category, Her2 status, tumor grading) and survival. EBER positivity was observed in 14 cases. None of the EAC were positive for EBER. In contrast, we observed EBER positivity in 2/73 adenocarcinomas of the GEJ (2.7%) and 12/274 GC (4.4%). These were of intestinal type (seven cases) or unclassifiable (six cases), while only one case was of diffuse type according to the Lauren classification. No association between EBV and pT, pN, or tumor grading was found, neither was there a correlation with clinical outcome. None of the EBER positive cases were Her2 positive. In conclusion, EBV does not seem to play a role in the carcinogenesis of EAC. Moreover, adenocarcinomas of the GEJ show lower rates of EBV positivity compared to GC. Our data only partially correlate with previous reports from the literature. This highlights the need for further research on this distinct entity. Recent reports, however, have identified specific epigenetic and genetic alterations in EBV-associated GC, which might lead to a distinct treatment approach for this specific subtype of GC in the future.

## Introduction

The presence of Epstein–Barr virus (EBV) in a subset of gastric carcinomas (GC) was first reported in 1990 (1). Today, it is recognized that EBV-associated GC show distinct molecular alterations suggesting a specific tumorigenesis pathway. Morphologically, EBV positivity was first described in lymphoepithelioma-like GC. In this particular histological subtype, the prevalence of EBV positivity is observed in more than 90% of cases (2–4). EBV can also be detected in carcinomas with conventional histology, namely, with diffuse or intestinal type according to the Lauren classification and all show an increased amount of tumor infiltrating lymphocytes (2, 4, 5).

Although approximately 5–20% of GC are found to be associated with EBV (2, 4, 6, 7), the prevalence of EBV in other gastrointestinal adenocarcinomas of the upper gastrointestinal tract, such as esophageal adenocarcinomas (EAC) and adenocarcinomas of the gastro-esophageal junction (GEJ) seems to be far less important (8, 9).

In this paper, we report our experiences on the role, frequency, and possible prognostic and biologic impact of EBV in gastro-esophageal adenocarcinomas in collections of primary resected tumors from two surgical centers and discuss our findings in the context of the current state of research in this field.

## Materials and Methods

### Patients and tissues

Formalin-fixed, paraffin-embedded (FFPE) archival cancer tissue from 118 patients with EAC (i.e., AEG I according to Siewert) (10), 73 patients with adenocarcinomas of GEJ (i.e., AEG II and AEG III according to Siewert) (10), and 274 patients with GC who underwent primary surgery between 1995 and 2005 at the Klinikum Rechts der Isar of the Technische Universität München, Germany (GEJ and GC), and between 1990 and 2011 at the Inselspital Bern, Switzerland (EAC). None of the patients had received pre- or perioperative neoadjuvant treatment. TNM-staging was performed according to the UICC/AJCC system 7th edition (11) and histopathological grading was done in accordance to the WHO (12). The pathologic features of the case collections are given in Table 1. Follow-up data (overall survival) were available from 397 patients. The use of archival tissue for research was approved by the local ethical commissions.

**Table 1:** **Comparison of clinicopathological characteristics of esophageal adenocarcinomas, adenocarcinomas of the gastro-esophageal junction and gastric carcinomas**.

		EAC	GEJ	GC
Gender	Male	102	51	170
	Female	16	22	104
pT category	pT1	36	4	20
	pT2	26	5	26
	pT3	51	29	84
	pT4	5	35	144
pN category	pN0	58	18	67
	pN1	19	27	82
	pN2	22	19	95
	pN3	19	9	30
Distant metastases	Absent	113	61	198
	Present	5	12	76
Grading	G1	18	0	1
	G2	51	12	42
	G3	49	61	231
Lauren classification	Intestinal	101	40	112
	Mixed	12	12	48
	Diffuse	3	16	96
	Non-classifiable	2	5	18

*EAC, esophageal adenocarcinomas; GEJ, gastro-esophageal junction; GC, gastric carcinomas*.

### EBER *in situ* hybridization

EBV-encoded small RNAs (EBER) *in situ* hybridization for the detection of EBV infection was performed as described before (13) and applied on tissue microarrays (TMA). The TMAs have already been used in several studies (GEJ and GC cases) (14) or were recently constructed (EAC cases). The TMAs contained three 1.0 mm cores per case (GEJ, GC) or six 0.6 mm cores per case (EAC), each from different tumor regions.

Freshly cut (3 μm) slides were deparaffinized, and endogenous peroxidase activity was quenched by incubation in 1% H_2_O_2_ in methanol. Slides were washed in ethanol and air-dried. The sections were incubated with an EBER probe (DAKO Cytomations, Glostrup, Denmark) for 90 min at 55°C or with PBS for negative controls. Immunodetection was then performed with the Labvision (Labvision, Fremont, CA, USA) detection system and visualized with 5-bromo-4-chloro-3-indolyl-phosphate. For negative controls, the EBER probe was omitted.

### Her2 analysis (immunohistochemistry and fluorescence *in situ* hybridization)

Data for Her2 in GC and adenocarcinomas of the GEJ were obtained from a previous study (14). In brief, for FISH analysis, an assay with fluorescence-labeled locus-specific DNA probes for Her2 and chromosome-17 (CEP17) centromeric α-satellite (Chrombios) was hybridized onto 4 μm TMA sections. The evaluation of FISH signals was performed by visual counting using an epifluorescence microscope (Zeiss Axioplan, Carl Zeiss Microimaging GmbH) according to current recommendations (15). Amplification was diagnosed when Her2/CEP17 quotient was >2.

For immunohistochemistry, dewaxed and rehydrated slides were incubated with an antibody against Her2 (DAKO, Glostrup, DK), following heat-induced antigen retrieval using 10 mm citrate buffer, pH 6, H_2_O_2_ blocking using 3% H_2_O_2_ in distilled water and avidin biotin blocking (Avidin/Biotin blocking kit, Vector Laboratories, Inc., Burlingame, CA, USA). Positive and negative controls were included in each reaction. Assessment of Her2 expression by immunohistochemistry (scores 0 to 3+) was done according to published recommendations (16). Her2 status was defined as Her2 3+ immunoreaction or Her2 2+ with additional detection of amplification by FISH (according to the EMEA/FDA criteria) (17).

### Statistical analysis

For statistical analysis, IBM SPSS 21.0 Statistics statistical software (SPSS Inc., Chicago, IL, USA) was used. Associations between EBER-ISH and pathological features as well as immunohistochemical expression patterns and FISH results, respectively, were given in crosstabs and were evaluated with *X*^2^ and Fisher’s exact tests. Survival analysis was done using Kaplan–Meier estimates and log rank tests. All tests were two-sided, and the significance level was set at 0.05.

## Results

### EBV detection in gastro-esophageal adenocarcinomas

EBV-encoded small RNAs positivity was observed in 14 cases in total. None of the EAC were positive for EBER. In contrast, we observed EBER positivity in 2/73 adenocarcinomas of the GEJ (2.7%) and 12/274 GC (4.4%). One case of GC showed EBER positivity only in the accompanying lymphocytic infiltrate but not in the tumor cells. In positive cases, all tumor cells showed positive staining, and showed no intratumoral heterogeneity across the different TMA cores.

### Correlation with clinic-pathologic features

**Figure 1:**
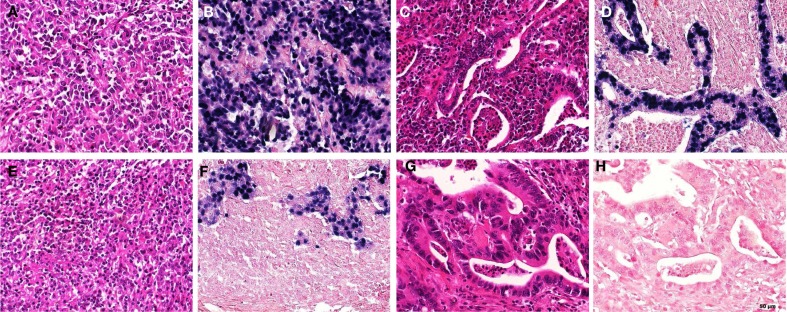
**Examples of EBER staining patterns and morphology in gastric carcinomas**. **(A,B)** “Lymphoepithelioma-like” morphology; EBER positive [**(A)** HE; **(B)** EBER-ISH], **(C,D)** “intestinal type” morphology, EBER positive [**(C)** HE; **(D)** EBER-ISH]; **(E,F)** “lymphoepithelioma-like” morphology; EBER negative in the tumor, but positive in accompanying lymphocytic infiltrate [**(E)** HE; **(F)** EBER-ISH]; **(G,H)** “intestinal type” morphology, EBER negative [**(G)** HE; **(H)** EBER-ISH] (EBER, EBV-encoded small RNAs; ISH, *in situ* hybridization).

EBV-encoded small RNAs positive cases were of intestinal type (seven cases) according to the Lauren classification, while only one case was of diffuse type. Six cases were unclassifiable according to the Lauren classification, but these tumors showed the characteristic lymphoepithelioma-like carcinoma morphology. Interestingly, the one EBV negative case with the EBV positive lymphoid infiltrate showed this particular pattern as well. Selected examples of EBV positive GC are shown in Figure 1.

An association between EBV and pT category, pN category, or tumor grading was not found neither was there any correlation with patient’s clinical outcome.

The pathologic features of all EBER positive cases are given in Table 2.

**Table 2:** **Clinicopathological characteristics of all EBER positive cases**.

Patient	Gender	Age	Local	pT	pN	pM	R-status	Grading	Lauren class
1	F	70	GEJ	3	0	0	0	3	Intestinal
2	M	70	GEJ	4	4	0	1	2	Intestinal
3	M	75	GC	1	0	0	0	2	Intestinal
4	M	84	GC	2	0	0	0	3	Non-classifiable
5	F	85	GC	1	0	0	0	3	Non-classifiable
6	M	63	GC	1	1	0	0	3	Intestinal
7	F	78	GC	3	1	0	0	3	Non-classifiable
8	M	52	GC	3	2	0	0	3	Intestinal
9	M	64	GC	4	3	1	2	3	Intestinal
10	M	68	GC	4	3	0	1	3	Diffuse
11	M	42	GC	4	3	1	2	3	Non-classifiable
12	M	50	GC	3	3	0	0	3	Non-classifiable
13	F	75	GC	4	4	0	0	3	Intestinal
14	F	74	GC	3	4	0	0	3	Non-classifiable

*GEJ, gastro-esophageal junction; GC, gastric carcinomas; Local, localization; R-status, resection status; EBER, EBV-encoded small RNAs*.

### EBV and Her2 in gastric carcinomas and adenocarcinomas of the esophagogastric junction

Results of the EBER analysis for GC and adenocarcinomas of the GEJ were also compared with data for Her2 expression and amplification from a previous study (see above). Her2 data were available from 336 cases. Of these, 36 tumors (10.7%) were classified as Her2 positive according to the FDA criteria described above. The EBER positive tumors were all negative for Her2 (Table 3).

**Table 3:** **Association between Her2 and Epstein–Barr virus in gastric cancer**.

		Her2 status		Total
		Negative	Positive	
EBV	Negative	286	36	322
	Positive	14	0	14
Total		300	36	336

## Discussion

### Prevalence of EBV in gastro-esophageal cancer

Since its first description, EBV-associated GC has emerged as a distinct subtype of GC, with an average prevalence of almost 10%. The frequency of detection of EBV, however, varies between 5 and 20%, which may depend on the patient collections in different studies (2, 4, 6, 7). Geographic differences are discussed as a reason for this variation, although not confirmed in meta-analysis (4). Of interest, EBV-associated tumorigenesis seems to be rather restricted to gastric cancer whereas the role of EBV in other gastrointestinal carcinomas such as esophageal carcinomas or small and large bowel cancers (9, 13, 18) seems to be negligible.

In the present study, we observed EBV positivity determined by EBER *in situ* hybridization in 14/465 cases of upper gastrointestinal adenocarcinomas in total. Of interest, none of the EAC were positive for EBER. This is in line with previous reports where there was no EBV positivity detected in collections with EAC (8) or squamous cell carcinomas (9) using EBER *in situ* hybridization. The rate of EBER positivity was very low in adenocarcinomas of the GEJ as well. In true GC, we could demonstrate EBER positivity in 4.4%, with a predominance of distal carcinomas (10 cases out of 12). The rate of positive cases in total is lower than expected from the literature. EBER *in situ* hybridization is recommended as a sensitive and specific method of choice for the detection of EBV in human tissue and tumors (2) and is usually used for the detection of EBV. Comparable to other studies, we used a TMA approach. We furthermore had two separate staining reactions for every case and there was no intratumoral heterogeneity in the staining patterns, therefore we regard our results of ISH as true findings. EBER-ISH furthermore allows the accurate localizing of the infected cells and we clearly could demonstrate the EBER positivity in the tumor cells. However, we also observed one case with lymphoepithelioma-like morphology and EBER positivity in the accompanying lymphocytic infiltrate but not in the carcinoma cells. Such a finding may explain why some studies have reported higher false positive rates, namely by interpreting DNA or RNA results based on extracts from whole slides containing both tumor and lymphatic tissue rather than exclusively tumor tissue (19, 20).

### Morphology of EBV-associated gastric cancer

Epstein–Barr virus positivity was first described in lymphoepithelioma-like GC, where its prevalence is extremely high (over 90%) (2–4). EBV can also be detected in carcinomas with conventional histology. Moreover, a higher proportion of diffuse versus intestinal type of GC according to Lauren classification has been reported in several studies. Nonetheless, no significant difference regarding other clinic-pathologic features could be found between intestinal or diffuse patterns in meta-analysis (2, 4). In contrast, in our study from a Western population, we found comparable numbers of EBV positive solid, lymphoepithelioma-like carcinomas (unclassifiable according to the Lauren classification) and conventional, intestinal type carcinomas, which morphologically did not significantly differ from other intestinal type tumors. In routine diagnostic setting, the detection of EBV-associated GC may therefore be hampered by the lack of a specific histologic appearance. In view of potential therapeutic consequences, which will be discussed later, a broader usage of EBV testing might be warranted in a future diagnostic workup of GC.

### Molecular genetics of EBV-associated gastric cancer

It has been shown that the virus remains in an episomal location in the monoclonal infected tumor cells and that EBER are abundantly expressed. As already discussed, the gold standard for specific detection of GC bearing an EBV infection is the performance of EBER *in situ* hybridization (2, 5).

The entry of EBV into epithelial cells is more complicated and inefficient compared to the similar process in B-lymphocytes. In B-cells, the virus utilizes binding of gp 42 to the human leukocyte antigen class II, whereas epithelial cells lacking HLA class II require more complex ways involving integrin complexes to reach the intracellular compartment. Despite those differences, the virus is able to infect both epithelium and lymphocytes (21–23).

Epstein–Barr virus-associated tumors are strongly correlated with methylation of CpG islands in the promoter region of cancer-related genes as well as with genome-wide hypermethylation, whereas microsatellite instable (MSI) tumors show other hypermethylation patterns, suggesting a different mechanism leading to epigenetic dysregulation in EBV and MSI tumors (24–27). This assumption is supported by the fact that EBV positivity in GC has been shown to be mutually exclusive to loss of MLH1 expression and MSI (28).

In a recent publication, a division into five molecular subtypes of gastric cancer based on findings of whole genome sequencing has been proposed. In addition to the well-known histomorphological subtypes according to the Lauren classification (intestinal, diffuse, and mixed type), two supplementary molecular subtypes, MSI and EBV, have been included. Furthermore, the authors confirm the epigenetic differences between EBV-associated and MSI GC. The former display low levels of demethylation and extensive genome-wide hypermethylation, whereas the latter is characterized on the other hand by broad demethylation and less hypermethylation (28). The identification of this specific molecular genetic pattern of EBV positive GC was also confirmed in a second very recent study of the cancer genome atlas (TCGA) project (29): based on their findings the authors propose a molecular classification by dividing gastric cancer into four subtypes (EBV positive, MSI, genomically stable tumors, and chromosomal instable tumors). EBV positive carcinomas show recurrent PIK3CA mutations, extreme DNA hypermethylation, and amplification of JAK2, CD274, and PDCD1LG2. Aberrations of the latter molecules (also known as PD-L1 and PD-L2) may not only serve for a molecular genetic classification but also as specific targets for immunotherapy (30, 31). Based on these results, testing for EBV might be warranted for GC in a future diagnostic setting.

Another potential therapeutically relevant marker, Her2, which is overexpressed or amplified in a significant subset of gastro-esophageal adenocarcinomas and which is regarded as a predictive marker for anti-Her2 targeted therapy (32), has been shown to be less expressed in EBV-associated GC compared to EBV negative GC (33–36). Supplementary to this data, which originate from Asian patient collectives, we could not detect any Her2 positive cases in the group of EBV-associated carcinomas. Our study is the first to analyze the correlation of Her2 and EBV in gastric cancer in a Western population. The reliability of our data was moreover increased by assessing the Her2 status according to the EMEA/FDA guidelines.

### EBV-associated gastric cancer and clinic-pathologic features

In EBV positive GC, the proportion of male gender has reported to be significantly higher in contrast to female patients (2, 4, 7), which we also could observe in our case collection. The prognostic relevance of EBV infection in GC has not been clearly elucidated so far. Most studies describe a favorable prognosis with improved survival and lower rate of lymph node metastases in patients with EBV-associated GC compared to EBV negative GC. A recent meta-analysis supports these findings, reporting lower tumor and lymph node stages according to TNM classification as well as a lower rate of distant metastases. EBV positivity was further associated with lower mortality rate when adjusted for stage and other confounders (37). However, another meta-analysis exhibited contradictory results, reporting no association of EBV positivity with depth of tumor invasion, lymph node metastases, or clinical stage (2), which is in line with our observations: we could not detect an association between EBV and pT category, pN category, or tumor grading, neither was there a correlation with clinical outcome of the patients in our case collection.

The EBV status of carcinoma cells does not influence current therapeutic schemes (7), but the recent molecular genetic findings may raise the question whether EBV positivity could act as a predictive marker in GC and lead to novel therapeutic options. Indeed, in different studies EBV positive GC were associated with chemoresistance against various cytotoxic drugs (38, 39). Since our case collection consisted of primary resected tumors only, we could not demonstrate any correlation between tumor regression and EBV status in the present study. However, there are data about the successful use of specific drug combinations in order to overcome the resistance to conventional chemotherapeutics, which also might influence neoadjuvant or other multimodal therapy concepts (38, 40). The mechanisms involved in this process need to be better understood, in order to implement specific treatment options for this subgroup of GC.

## Summary

In conclusion, we could demonstrate the presence of EBV by EBER *in situ* hybridization in a subset of gastric cancers and also in a small number of adenocarcinomas of the GEJ. In contrast, EBV does not seem to play a role in the carcinogenesis of EAC. However, the data from our case collections only partially correlate with previous reports from the literature. This highlights the need for further research on this distinct entity. The pathogenetic role of EBV in carcinogenesis is still poorly understood and the presence of EBV has no therapeutic implication at present. Most recent reports, however, identified different genetic and epigenetic alterations in EBV-associated GC compared to viral negative GC. Targeting the viral infection itself or molecules deregulated within this specific molecular background might lead to a distinct treatment approach in future perspectives and might also influence routine diagnosis of gastric cancer with regard to detection of EBV positive cases.

## Conflict of Interest Statement

The authors declare that the research was conducted in the absence of any commercial or financial relationships that could be construed as a potential conflict of interest.

## References

[1] Burke AP Yen TS Shekitka KM Sobin LH. Lymphoepithelial carcinoma of the stomach with Epstein-Barr virus demonstrated by polymerase chain reaction. Mod Pathol (1990) 3:377–80. 2163534

[2] Lee JH Kim SH Han SH An JS Lee ES Kim YS. Clinicopathological and molecular characteristics of Epstein-Barr virus-associated gastric carcinoma: a meta-analysis. J Gastroenterol Hepatol (2009) 24:354–65. 10.1111/j.1440-1746.2009.05775.x 19335785

[3] Bittar Z Fend F Quintanilla-Martinez L. Lymphoepithelioma-like carcinoma of the stomach: a case report and review of the literature. Diagn Pathol (2013) 8:184. 10.1186/1746-1596-8-184 24188515 PMC4228252

[4] Murphy G Pfeiffer R Camargo MC Rabkin CS. Meta-analysis shows that prevalence of Epstein-Barr virus-positive gastric cancer differs based on sex and anatomic location. Gastroenterology (2009) 137:824–33. 10.1053/j.gastro.2009.05.001 19445939 PMC3513767

[5] Fukayama M Ushiku T. Epstein-Barr virus-associated gastric carcinoma. Pathol Res Pract (2011) 207:529–37. 10.1016/j.prp.2011.07.004 21944426

[6] Kijima Y Hokita S Takao S Baba M Natsugoe S Yoshinaka H Epstein-Barr virus involvement is mainly restricted to lymphoepithelial type of gastric carcinoma among various epithelial neoplasms. J Med Virol (2001) 64:513–8. 10.1002/jmv.1079 11468737

[7] Nishikawa J Yoshiyama H Iizasa H Kanehiro Y Nakamura M Nishimura J Epstein-Barr virus in gastric carcinoma. Cancers (2014) 6:2259–74. 10.3390/cancers6042259 25386788 PMC4276965

[8] Sarbia M zur Hausen A Feith M Geddert H von Rahden BH Langer R Esophageal (Barrett’s) adenocarcinoma is not associated with Epstein-Barr virus infection: an analysis of 162 cases. Int J Cancer (2005) 117:698–700 10.1002/ijc.21190 15929074

[9] Cho YJ Chang MS Park SH Kim HS Kim WH. In situ hybridization of Epstein-Barr virus in tumor cells and tumor-infiltrating lymphocytes of the gastrointestinal tract. Hum Pathol (2001) 32:297–301. 10.1053/hupa.2001.22766 11274639

[10] Siewert JR Stein HJ Classification of adenocarcinoma of the oesophagogastric junction. Br J Surg (1998) 85:1457–9 10.1046/j.1365-2168.1998.00940.x 9823902

[11] Sobin LH Gospodarowicz MK Wittekind C TNM Classification of Malignant Tumors. 7th ed Oxford: Wiley-Blackwell (2009).

[12] Bosman FT Carneiro F Hruban RH Theise ND WHO Classification of Tumours of the Digestive System. Lyon: IARC (2010).

[13] von Rahden BH Langner C Brücher BL Stein HJ Sarbia M. No association of primary adenocarcinomas of the small bowel with Epstein-Barr virus infection. Mol Carcinog (2006) 45:349–52. 10.1002/mc.20163 16493667

[14] Berezowska S Novotny A Bauer K Feuchtinger A Slotta-Huspenina J Becker K Association between HSP90 and Her2 in gastric and gastroesophageal carcinomas. PLoS One (2013) 8(7):e69098. 10.1371/journal.pone.0069098 23874879 PMC3708885

[15] Rauser S Weis R Braselmann H Feith M Stein HJ Langer R Significance of HER2 low-level copy gain in Barrett’s cancer: implications for fluorescence in situ hybridization testing in tissues. Clin Cancer Res (2007) 13:5115–23. 10.1158/1078-0432.CCR-07-0465 17785566

[16] Rüschoff J Hanna W Bilous M Hofmann M Osamura RY Penault-Llorca F HER2 testing in gastric cancer: a practical approach. Mod Pathol (2012) 25:637–50. 10.1038/modpathol.2011.198 22222640

[17] Assessment Report for Herceptin. Procedure No. EMEA/H/C/278/II/0047 [Internet]. London: European Medicines Agency (2010) [cited 2015 Feb 15]. Available from: http://www.ema.europa.eu/docs/en_GB/document_library/EPAR_-_Assessment_Report_-_Variation/human/000278/WC500074921.pdf

[18] Wong NA Herbst H Herrmann K Kirchner T Krajewski AS Moorghen M Epstein-Barr virus infection in colorectal neoplasms associated with inflammatory bowel disease: detection of the virus in lymphomas but not in adenocarcinomas. J Pathol (2003) 201:312–8. 10.1002/path.1442 14517849

[19] Awerkiew S Bollschweiler E Metzger R Schneider PM Hölscher AH Pfister H. Esophageal cancer in Germany is associated with Epstein-Barr-virus but not with papillomaviruses. Med Microbiol Immunol (2003) 192:137–40. 10.1007/s00430-002-0128-z 12920588

[20] Awerkiew S zur Hausen A Baldus SE Hölscher AH Sidorenko SI Kutsev SI Presence of Epstein-Barr virus in esophageal cancer is restricted to tumor infiltrating lymphocytes. Med Microbiol Immunol (2005) 194:187–91. 10.1007/s00430-004-0233-2 15692828

[21] Borza CM Hutt-Fletcher LM. Alternate replication in B cells and epithelial cells switches tropism of Epstein-Barr virus. Nat Med (2002) 8:594–9. 10.1038/nm0602-594 12042810

[22] Tsao SW Tsang CM To KF Lo KW. The role of Epstein-Barr virus in epithelial malignancies. J Pathol (2015) 235(2):323–33. 10.1002/path.4448 25251730 PMC4280676

[23] Hutt-Fletcher LM Epstein-Barr virus entry. J Virol (2007) 81:7825–32 10.1128/JVI.00445-07 17459936 PMC1951282

[24] Kusano M Toyota M Suzuki H Akino K Aoki F Fujita M Genetic, epigenetic, and clinicopathologic features of gastric carcinomas with the CpG island methylator phenotype and an association with Epstein-Barr virus. Cancer (2006) 106:1467–79. 10.1002/cncr.21789 16518809

[25] Chang MS Uozaki H Chong JM Ushiku T Ishikawa S Hino R CpG island methylation status in gastric carcinoma with and without infection of Epstein-Barr virus. Clin Cancer Res (2006) 12:2995–3002 10.1158/1078-0432.CCR-05-1601 16707594

[26] Zong L Seto Y. CpG island methylator phenotype, *Helicobacter pylori*, Epstein-Barr virus, and microsatellite instability and prognosis in gastric cancer: a systematic review and meta-analysis. PLoS One (2014) 9:e86097. 10.1371/journal.pone.0086097 24475075 PMC3903497

[27] Wang K Yuen ST Xu J Lee SP Yan HH Shi ST Whole-genome sequencing and comprehensive molecular profiling identify new driver mutations in gastric cancer. Nat Genet (2014) 46:573–82. 10.1038/ng.2983 24816253

[28] Park HY Kang SY Kang GH Bae GE Lee SE Kim KM EBV infection and mismatch repair deficiency mediated by loss of hMLH1 expression contribute independently to the development of multiple synchronous gastric carcinomas. J Surg Oncol (2012) 106:777–82. 10.1002/jso.23131 22513802

[29] Cancer Genome Atlas Research Network. Comprehensive molecular characterization of gastric adenocarcinoma. Nature (2014) 513:202–9. 10.1038/nature13480 25079317 PMC4170219

[30] McDermott DF Atkins MB. PD-1 as a potential target in cancer therapy. Cancer Med (2013) 2:662–73. 10.1002/cam4.106 24403232 PMC3892798

[31] Ohaegbulam KC Assal A Lazar-Molnar E Yao Y Zang X. Human cancer immunotherapy with antibodies to the PD-1 and PD-L1 pathway. Trends Mol Med (2014) 21(1):24–33. 10.1016/j.molmed.2014.10.009 25440090 PMC4282825

[32] Bang YJ Van Cutsem E Feyereislova A Chung HC Shen L Sawaki A Trastuzumab in combination with chemotherapy versus chemotherapy alone for treatment of HER2-positive advanced gastric cancer or gastro-oesophageal junction cancer (ToGA): a phase 3, open-label, randomised controlled trial. Lancet (2010) 376:687–97. 10.1016/S0140-6736(10)61121-X 20728210

[33] Zhang YW Zhao XX Tan C Zhang ZG Jiang Y Chen JN Epstein-Barr virus latent membrane protein 2A suppresses the expression of HER2 via a pathway involving TWIST and YB-1 in Epstein-Barr virus-associated gastric carcinomas. Oncotarget (2014) 6(1):207–20. 25402957 10.18632/oncotarget.2702 PMC4381589

[34] Sukawa Y Yamamoto H Nosho K Kunimoto H Suzuki H Adachi Y Alterations in the human epidermal growth factor receptor 2-phosphatidylinositol 3-kinase-v-Akt pathway in gastric cancer. World J Gastroenterol (2012) 18:6577–86. 10.3748/wjg.v18.i45.6577 23236232 PMC3516204

[35] Song HJ Srivastava A Lee J Kim YS Kim KM Ki Kang W Host inflammatory response predicts survival of patients with Epstein-Barr virus-associated gastric carcinoma. Gastroenterology (2010) 139:84–92. 10.1053/j.gastro.2010.04.002 20398662

[36] Lee HS Chang MS Yang HK Lee BL Kim WH. Epstein-Barr virus-positive gastric carcinoma has a distinct protein expression profile in comparison with Epstein-Barr virus-negative carcinoma. Clin Cancer Res (2004) 10:1698–705. 10.1158/1078-0432.CCR-1122-3 15014022

[37] Camargo MC Kim WH Chiaravalli AM Kim KM Corvalan AH Matsuo K Improved survival of gastric cancer with tumour Epstein-Barr virus positivity: an international pooled analysis. Gut (2013) 63:236–43. 10.1136/gutjnl-2013-304531 23580779 PMC4384434

[38] Shin JY Kim JO Lee S Chae HS Kang JH. LY294002 may overcome 5-FU resistance via down-regulation of activated p-AKT in Epstein-Barr virus-positive gastric cancer cells. BMC Cancer (2010) 10:425. 10.1186/1471-2407-10-425 20704765 PMC3087326

[39] Banerjee AS Pal AD Banerjee S. Epstein-Barr virus-encoded small non-coding RNAs induce cancer cell chemoresistance and migration. Virology (2013) 443:294–305. 10.1016/j.virol.2013.05.020 23791019

[40] Seya T Tanaka N Yokoi K Ishikawa N Horiba K Kanazawa Y Complete response of a patient with advanced gastric cancer, showing Epstein-Barr virus infection, to preoperative chemotherapy with S-1 and cisplatin. Int J Clin Oncol (2007) 12:472–7. 10.1007/s10147-007-0682-x 18071868

